# Blount's disease: The importance of early diagnosis and early treatment

**DOI:** 10.1002/ccr3.2214

**Published:** 2019-05-20

**Authors:** Ioannis Delniotis, Benedikt Leidinger, Aikaterini Kyriakou, Nikiforos Galanis

**Affiliations:** ^1^ Department of Orthopaedics Papageorgiou General Hospital, Medical School, Aristotle University of Thessaloniki Thessaloniki Greece; ^2^ Department of Paediatric Orthopaedics – Foot & Ankle Surgery Orthopaedic Clinic Volmarstein Wetter (Ruhr) Germany; ^3^ 2nd Department of Dermatology Papageorgiou General Hospital, Medical School, Aristotle University of Thessaloniki Thessaloniki Greece

**Keywords:** Blount's disease, early diagnosis, early treatment, genu varum

## Abstract

The purpose of this image is to raise awareness of the early diagnosis and treatment of Blount's disease. Failure to distinguish between physiologic genu varum and early onset of Blount's disease can lead to irreversible outcomes, including progressive deformity with gait deviations, limb‐length discrepancy, and premature arthritis of the knee.

## 1 HOW CAN I DISTINGUISH PHYSIOLOGIC GENU VARUM FROM EARLY ONSET OF BLOUNT'S DISEASE?

**Figure 1:**
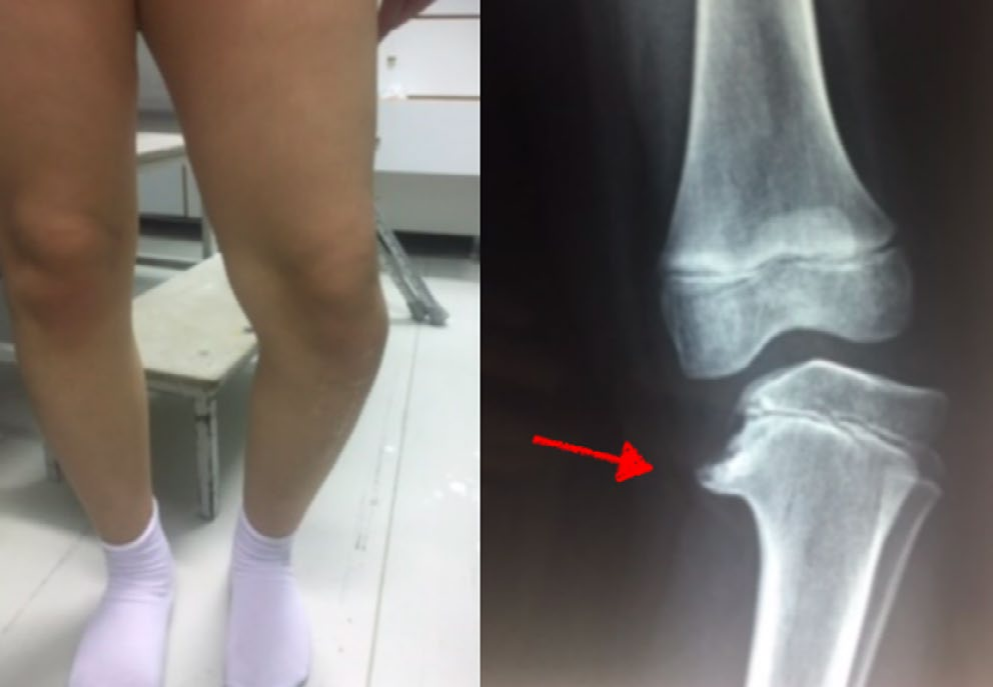
Clinical presentation of Blount's disease. The red arrow shows the “metaphyseal beaking,” suggestive of the disease

A 10‐year‐old female presented to our department for progressive bowing of the left tibia and significant limb‐length discrepancy (left lower limb shortened by approximately 1.5 cm; Figure 1). Although a significant varus deformity of her left leg began at age of 3 (early onset of Blount's disease), she was finally treated at age of 8 with a proximal tibia and fibula valgus osteotomy, fixed with a unilateral, uniplanar external fixator in the tibia. The surgery did not improve the patient's condition at all.

Genu varum is a normal physiologic process (bowed legs) in kids less than 2 years old. This situation changes to a neutral position by 18‐24 months of age and continues in order to reach a peak of genu valgum (knocked knees) in the age of 3‐4. Clinicians should retain a high index of suspicion when a child ≥3 years old presents with severe, asymmetric, varus deformity focused at proximal tibia. Radiologic findings that can help clinicians to distinguish between physiologic genu varum and cases of early onset of Blount's disease are the medial tibial metaphyseal “beaking” and fragmentation (Langenskiold classification I‐VI) and the tibial metaphyseal‐diaphyseal angle (<11 degrees physiologic bowing, >16 degrees consistent with Blount's disease and higher chance of progression).^1^

Good candidates for surgical treatment of the early onset of Blount's disease are patients >3 years and/or Langenskiold stages III‐VI. If this is not the case, then surgical treatment of neglected Blount's disease can be a real challenge for orthopedic surgeons and may require elevation of medial plateau, combined osteotomies, use of Taylor Spatial Frame or Ilizarov ring external fixation, and progressive limb‐lengthening. However, with careful, individualized, preoperative planning most patients with Blount's disease can expect very good functional and cosmetic results.^2^

## CONFLICT OF INTEREST

None declared.

## AUTHOR CONTRIBUTION

ID: drafted the manuscript, obtained the photographs, and contributed to patient care. BL: critically reviewed the paper. AK: revised the manuscript. NG: contributed to patient care and revised the manuscript.
